# Phylogenetic Identification, Phenotypic Variations, and Symbiotic Characteristics of the Peculiar Rhizobium, Strain CzR2, Isolated from *Crotalaria zanzibarica* in Taiwan

**DOI:** 10.1264/jsme2.ME16063

**Published:** 2016-09-29

**Authors:** Cheng-Tai Huang, Chi-Te Liu, Shiang-Jiuun Chen, Wen-Yuan Kao

**Affiliations:** 1 Institute of Ecology and Evolutionary Biology, National Taiwan University No. 1, Sec. 4Roosevelt Road, Taipei 106Taiwan; 2 Institute of Biotechnology, National Taiwan University R412., No. 81Chang-Xing St., Taipei, 106Taiwan; 3 Agricultural Biotechnology Research Center, Academia Sinica No. 128 Sec. 2Academia Rd., Nankang, Taipei, 115Taiwan; 4 Department of Life Science, National Taiwan University No. 1, Sec. 4Roosevelt Road, Taipei 106Taiwan

**Keywords:** *Bradyrhizobium arachidis*, *Crotalaria zanzibarica*, naturalized plant, multilocus sequence analysis (MLSA), legume-rhizobium symbiosis

## Abstract

*Crotalaria zanzibarica* is an exotic and widely distributed leguminous plant in Taiwan. The relationship between *C. zanzibarica* and its rhizobial symbionts has been suggested to contribute to its successful invasion. A rhizobial strain (designed as CzR2) isolated from the root nodules of *C. zanzibarica* and cultivated in standard YEM medium displayed pleomorphism, with cells ranging between 2 and 10 μm in length and some branching. In the present study, we identified this rhizobial strain, investigated the causes of pleomorphism, and examined the nodules formed. The results of a multilocus sequence analysis of the *atpD*, *dnaK*, *glnII*, *gyrB*, *recA*, and *rpoB* genes revealed that CzR2 belongs to *Bradyrhizobium arachidis*, a peanut symbiont recently isolated from China. Cells of the strain were uniformly rod-shaped in basal HM medium, but displayed pleomorphism in the presence of yeast extract, mannitol, or fructose. These results indicate that the morphology of CzR2 in its free-living state is affected by nutrient conditions. Several highly pleomorphic bacteroids enclosed in symbiosomes were frequently detected in FM and TEM observations of sections of the indeterminate nodules induced by CzR2; however, no infection thread was identified. Flow cytometric analyses showed that CzR2 cells in YEM medium and in the nodules of *C. zanzibarica* had two or more than two peaks in relative DNA contents, respectively, suggesting that the elongated cells of CzR2 in its free-living state occur due to a cell cycle-delayed process, while those in its symbiotic state are from genomic endo-reduplication.

Soil bacteria that establish symbiotic relationships with legumes and induce tumor-like, N_2_-fixing structures (nodules) on roots are referred to as rhizobia. Rhizobia have been shown to improve legume yields in agriculture (32). Most of the research conducted on legume-rhizobium symbiosis has focused on legume crops and model legumes, such as *Glycine*, *Pisum*, *Medicago*, and *Lotus*, whereas few studies have examined other legumes (5). Besides their agricultural benefits, rhizobia may also play a critical role in affecting legume distributions in natural ecosystems. Previous studies reported that the growth of exotic legumes was limited by the scarcity of compatible rhizobia when they were first introduced into new habitats (17, 22). In order to become successfully established, naturalized legumes (“naturalized plants” in this study are exotic plants that sustain populations over many generations without human interventions, as defined by Richardson *et al.* [23]) need to acquire symbionts from the newly occupied habitats. Although legumes are the most contributors of the naturalized plants of the world (20), studies on compatible rhizobia in their introduced regions and nodule development have been restricted to a few species.

The genus *Crotalaria*, which comprises approximately 600 species distributed in tropical and subtropical regions, is a member of the subfamily *Papilionoideae* and most of its members are native to Africa (19). Some species of *Crotalaria* have been introduced as green manure or fodder in some countries (19). Although *Crotalaria* is a large genus that has spread worldwide, few studies have examined its symbiotic rhizobia. Based on limited findings, *Bradyrhizobium* strains are the most common symbionts isolated from *Crotalaria* species (1, 10, 25). However, some non-classical rhizobial strains, such as *Methylobacterium* (30) and *Burkholderia (*10), have also been reported to nodulate *Crotalaria* plants.

*Crotalaria* was introduced into Taiwan more than 80 years ago, and is the largest genus of naturalized legumes on the island (37). Among the *Crotalaria* spp. introduced, *C. zanzibarica*, a perennial leguminous shrub native to Africa, has become the most widely distributed naturalized legume in Taiwan (37, 38). This plant, which is mainly distributed along roadsides, riverbanks, and in abandoned fields, commonly establishes symbiosis with rhizobia forming root nodules. Although *C. zanzibarica* has been evaluated as a species of wide distribution and the highest invasiveness among naturalized legumes in Taiwan (37, 38), the relationship between its distribution and symbiotic rhizobia has not yet been addressed. In a preliminary study, we grew *C. zanzibarica*, neither inoculated nor fertilized, in a greenhouse and found that some individuals formed root nodules and grew better than those without these structures. We subjected these nodulated plants to further analyses and found that their nitrogen contents (% dry weight) were 5.1±0.2 in leaves and 8.1±0.3 in nodules (mean±SD, *n*=6). The results prompted us to study the symbiotic relationship between the plant and its root-nodulating rhizobia.

A peculiar rhizobium (defined as CzR2), displaying pleomorphism in its free-living (cultured in YEM medium) and symbiotic states, was isolated from the root nodules of greenhouse-grown *C. zanzibarica*. Based on its slow-growing phenotype, we suspected that this isolate belongs to the genus *Bradyrhizobium*. Accordingly, we analyzed the 16S rRNA gene sequence and several housekeeping and symbiotic genes of this rhizobium in order to elucidate its species identity. We also investigated phenotypic variations, in particular the causes of pleomorphism in YEM medium, of this strain and nodule development in *C. zanzibarica* inoculated with this strain.

## Materials and Methods

### Isolation of rhizobia from root nodules of *C. zanzibarica*

The seeds of *C. zanzibarica* were collected from the field and germinated in pots filled with a mixture of peat soil (Kekkila, Finland), vermiculite, and perlite (5:2:2), which were then placed in the greenhouse of National Taiwan University (Taipei, Taiwan). Although seeds and culture soil were both sterilized before cultivation, some plants still formed root nodules in the greenhouse, but not in a laboratory growth chamber. The fresh nodules of two-month-old plants were collected and surface sterilized by immersion in 0.5% SDS for 1 min, then 70% ethanol for 5 min, and washed three times using sterile deionized-distilled water (DDW). A nodule suspension was obtained by crushing nodules in DDW and spreading onto YEM agar plates (g L^−1^: NaCl, 0.2; MgSO_4_·7H_2_O, 0.2; K_2_HPO_4_, 0.5; FeSO_4_·7H_2_O, 0.005; mannitol, 10.0; yeast extract, 0.4; agar, 15.0; pH was adjusted to 6) at 30°C. When putative rhizobial colonies were visible, a single colony was selected and checked for unity by repeated streaking on YEM agar plates. A pure culture was obtained and designated as strain CzR2. A reference strain, *Bradyrhizobium japonicum* USDA 110 was purchased from the Bioresource Collection and Research Center (BCRC; Hsinchu, Taiwan) for comparative purposes. Pure cultures of each strain were stored in 15% glycerol-YEM broth at –80°C.

### Molecular identification of strain CzR2

Total genomic DNA was extracted from the pure culture of strain CzR2 grown in YEM broth at the late exponential phase of growth. The extraction of DNA was performed using the Geneaid DNA Mini kit (Geneaid Biotech, New Taipei, Taiwan). 16S rRNA genes were amplified using bacterial universal primers (11).

The other six housekeeping genes (*atpD*, *dnaK*, *glnII*, *gyrB*, *recA*, and *rpoB* genes) and three symbiotic genes (*nodA*, *nodZ*, and *nifH*) were amplified using primers described previously (12, 18, 29). The sequencing primers of all genes were the same as those used for PCR amplification.

The aforementioned sequences and reference sequences of housekeeping genes were aligned using the ClustalW program (33). The phylogenetic identification of strain CzR2 was conducted by analyzing the 16S rRNA gene and combined *atpD*-*dnaK*-*glnIIgyrB*-*recA*-*rpoB* gene dataset. Maximum likelihood (ML) phylogenies and model tests were performed using the software MEGA version 6 (31). The best-fit models for ML analyses were T92+G+I for the 16S rRNA gene and GTR+G+I for the *atpD*-*dnaK*-*glnIIgyrB*-*recA*-*rpoB* gene dataset. The topology of the trees was evaluated by bootstrapping with 1,000 replications. All sequences obtained in this study were deposited in the GenBank database. The GenBank accession numbers of strain CzR2 generated in this study are KJ125399 (16S rRNA), KU315329 (*atpD*), KU000974 (*dnaK*), KU000975 (*glnII*), KU315331 (*gyrB*), KU001035 (*recA*), KU001095 (*rpoB*), KJ125402 (*nodA*), KJ135034 (*nodZ*), and KJ135031 (*nifH*).

### Cellular morphology and growth dynamics in YEM medium

Although we demonstrated that CzR2 belongs to a specific species after the molecular analysis (results of this study), the type strain of that species was not available when the experiments of this study were conducted. Thus, instead of using the type strain, a reference strain, *B. japonicum* USDA 110, was used for comparative purposes. CzR2 and the reference strain USDA 110 were both grown on YEM agar plates at 30°C for 7 d. A single colony of each strain was selected and stained with DAPI (4,6-diamidino-2-phenylindole; Sigma-Aldrich, St. Louis, MO, USA) at 50 μg mL^−1^ at 25°C for 10 min. Cell samples were examined under a microscope (BX51; Olympus, Tokyo, Japan) with a bright or fluorescent field according to the method described by Liu *et al.* (9). In order to measure cell sizes, at least ten random fields of view were photographed and approximately 1,000 cells in each sample were examined using ImageJ 1.48 software (NIH, Bethesda, MD, USA).

Regarding the construction of growth curves, strain CzR2 and USDA 110 were inoculated into 3 mL of fresh YEM broth (five replicates for each strain) and grown at 30°C, 200 rpm on a shaking table. Population growth was evaluated by counting the number of CFU mL^−1^ every 24 h and measuring OD_600_ every 8 to 10 h in the broth. Cell size was also measured every 24 h during the culture period. Mean generation times were calculated from the exponential phase of growth based on OD_600_ measurements.

### Effects of culture substrates on phenotypes

HM (HEPES-MES) medium (2) was employed as basal medium to test the effects of substrates on the phenotypes of the rhizobial strains (CzR2 and USDA 110). Rhizobial strains were grown on plates containing HM basal medium only and HM basal medium in combination with yeast extract (0.04 or 0.3%), mannitol (0.5%), fructose (0.5%), or glucose (0.5%) at 30°C for 7 d.

### Observations of development, anatomy, and bacteroids of CzR2-infected nodules

The seeds of *C. zanzibarica* were sterilized using 0.5% SDS and 10 mM NaCl for 5 min, 70% ethanol for 30 min, and then rinsed with sterile water. These seeds were subsequently drilled using a sterilized needle to break the seed coat and germinated in a Petri dish at room temperature for 2 d. After germination, seedlings were transferred to 1-L glass pots filled with a sterilized mixture of peat soil, vermiculite, and perlite at a proportion of 5:2:2, respectively, and regularly irrigated with DDW. After 4–5 d of transplanting, plants in pots were inoculated either with 600 μL of strain CzR2 at the exponential phase in YEM broth or with YEM liquid medium without a rhizobial culture as a negative control. All seedlings were grown in a humidity-controlled (70%) growth chamber with 12 h of light (photosynthetic active radiation of 100–150 μmol m^−2^ s^−1^, at 28°C) and 12 h of dark (at 25°C). When plants were 40-d-old, they were transferred to 2-L plastic pots and placed in a glasshouse under natural daylight.

In histological observations, branched nodules from 2-month-old plants were excised, fixed by FPGA (formalin:propionic acid:95% glycerol:ethanol:H_2_O=1:1:3:7:8), and sectioned to a thickness of 15 μm using the microslicer DTK-1000 (Ted Pella, Redding, CA, USA). Sections were stained with 1% toluidine blue O (TBO), and then examined by light microscopy.

In fluorescence microscopic (FM) observations, bacteroids were extracted from fresh nodules (of 2-month-old plants) and stained with DAPI, following the aforementioned protocol. In transmission electron microscopic (TEM) observations, nodule samples were fixed with 2.5% glutaraldehyde, dehydrated in a graded series of acetone, and then embedded with Spurr’s resin. Ultrathin sections (thickness of 80 nm) were obtained using an ultramicrotome (PowerTome XL; RMC Boeckeler, Tucson, AZ, USA) and analyzed on a transmission electron microscope (H-7650; Hitachi, Tokyo, Japan) at 100 kV.

### DNA contents of free-living and symbiotic CzR2

The DNA contents of free-living cells and symbiotic bacteroids of CzR2 were assessed using flow cytometry. Free-living CzR2 was grown in YEM broth for 7 d and symbiotic bacteroids were extracted from the mature root nodules of *C. zanzibarica* (67-d-old). These cells were fixed in 90% ethanol at 20°C for 16 h, then washed twice with PBS (phosphate-buffered saline) followed by centrifugation at 4,000 rpm for 2 min. Pelleted cells were stained with propidium iodide (PI)-RNase staining buffer solution (BD Biosciences, San Jose, CA, USA) at room temperature for 30 min. Samples, at least 20,000 cells for each, were then analyzed with a Cytomics FC500 analyzer (Beckman Coulter, Brea, CA, USA). Data analyses were performed with CXP software (Beckman Coulter).

## Results

### Phylogenetic identification of strain CzR2

**Fig. 1:**
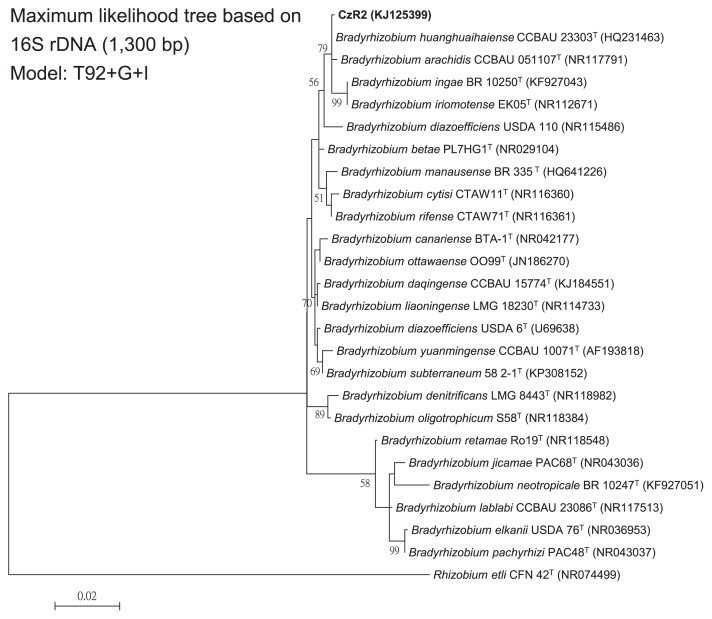
Phylogeny of strain CzR2 based on 16S rRNA genes. Maximum likelihood tree based on partial 16 rRNA genes (1,300 bp) showing the relationships among strain CzR2 and defined *Bradyrhizobium* species. Only bootstrap values >50 are shown at the internodes. The scale bar represents 2% nucleotide substitutions.

In the phylogenetic tree of the 16S rRNA gene (Fig. 1), strain CzR2 was grouped together with four *Bradyrhizobium* type strains, all of which had nearly identical sequences. Strain CzR2 showed 99.92% similarity to *B. huanghuaihaiense* CCBAU 23303^T^, 99.77% to *B. arachidis* CCBAU 51107^T^, and 99.39% to *B. iriomotense* EK05^T^ and *B. ingae* BR1025^T^.

**Fig. 2:**
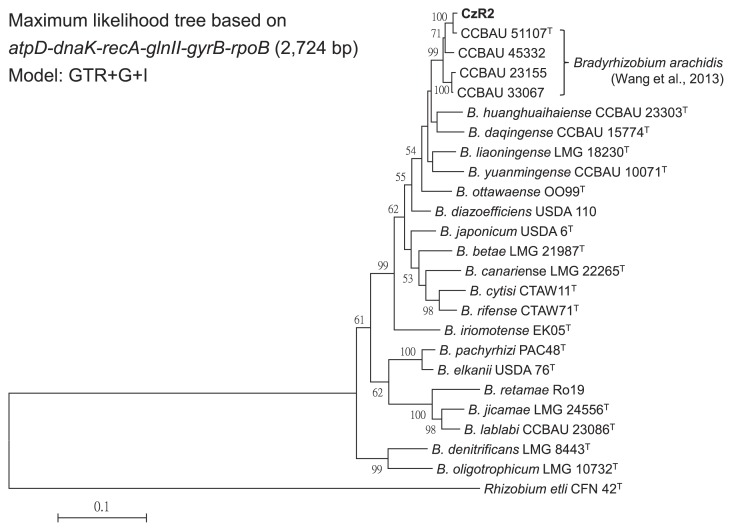
Phylogeny of strain CzR2 based on six housekeeping genes. The maximum likelihood tree based on concatenated *atpD*-*dnaK*-*glnII*-*gyrBrecA*-*rpoB* gene sequences (2,724 bp) showing the relationships among strain CzR2 and defined *Bradyrhizobium* species. Only bootstrap values >50 are shown at the internodes. The scale bar represents 10% nucleotide substitutions.

A ML analysis of the six concatenated housekeeping genes (*atpD*, *dnaK*, *glnII*, *gyrB*, *recA*, and *rpoB*) revealed that strain CzR2 and four CCBAU strains formed a well-supported group (Fig. 2). These four CCBAU strains were previously reported to belong to the novel species, *B. arachidis* (36). In a comparison of these six genes among the related strains, strain CzR2 was the most similar (99.27% identity) to the type strain of *B. arachidis*, CCBAU 51107, which is the peanut symbiont isolated from Hebei, China.

### Similarities in symbiotic genes between strain CzR2 and CCBAU 51107

Since the horizontal transfer of symbiotic genes is a common phenomenon in *Bradyrhizobium* lineages (1, 12, 18), we also compared symbiotic gene sequences between strain CzR2 and CCBAU 51107. These two strains had 98.71% similarity in the *nodA* gene (460 bp) and 99.85% similarity in the *nifH* gene (648 bp).

### Cellular morphology and growth dynamics of CzR2 and USDA 110 in YEM medium

**Fig. 3:**
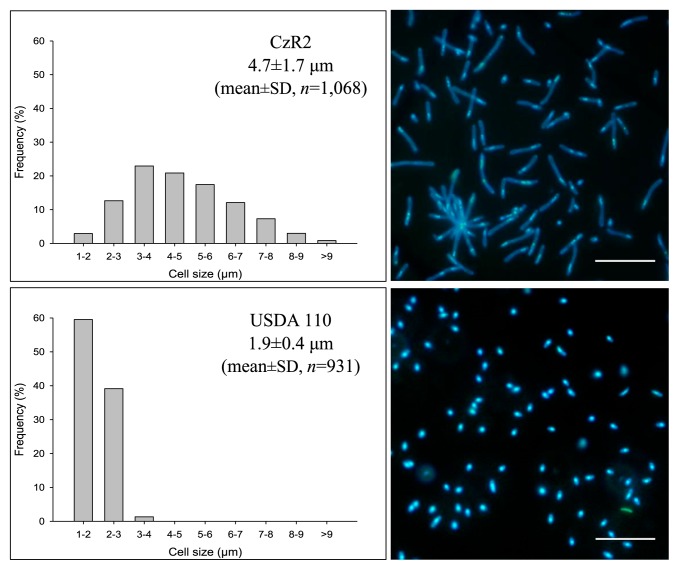
Cellular morphologies of strain CzR2 and USDA 110 on YEM plates. Size variations and distributions of strain CzR2 (Top) and USDA 110 (down) grown on YEM plate for 7 d. The pictures show these cells stained by DAPI. Bar, 10 μm.

Strain CzR2 and USDA 110 both formed detectable colonies on YEM plates after 5–6 d of being incubated, indicating a slow-growing phenotype. The cellular morphologies of these two strains were observed using optical microscopy. As shown in Fig. 3, the cell size of strain CzR2 varied (ranging between 1.4 and 10.9 μm in length) and branched cells were detected (1–2% of examined cells, data not shown). In addition, DNA was located at one end of the elongated or branched cells. In contrast, USDA 110 under the same growth conditions with CzR2 was uniformly rod-shaped and cell length ranged between 1.3 and 3.4 μm.

**Fig. 4:**
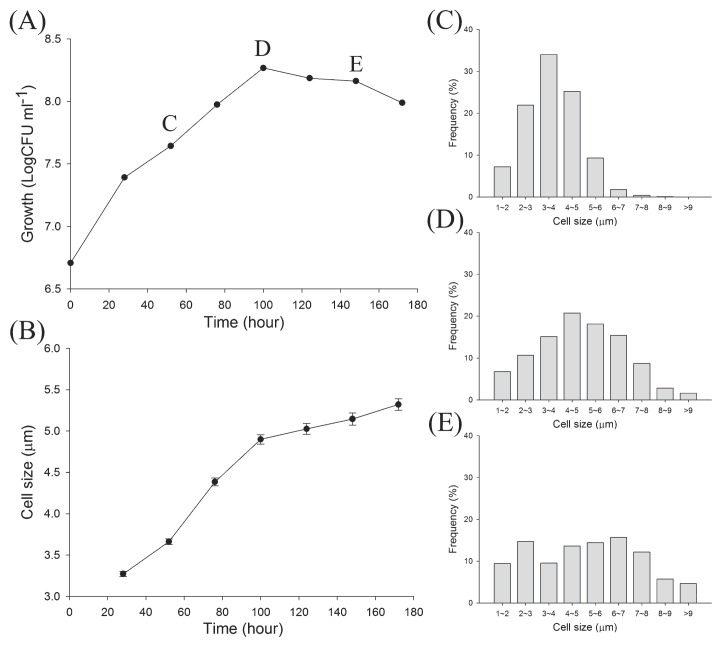
Cell size variations during growth kinetics of strain CzR2 grown in YEM broth. Growth was evaluated as the number of Log CFU mL^−1^ (A) and mean cell size (B). Error bars in (B) indicate standard errors (*n*>1,000). The distribution and frequency of cells with different sizes were also calculated in the exponential phase (C), transition phase (D), and stationary phase (E).

The mean length of the cells of strain CzR2 markedly increased from 3.2 to 5.0 μm during the exponential growth phase (Fig. 4), and approximately 3% of these cells were branched. In contrast, the cell size of USDA 110 remained unchanged, at approximately 2 μm in length, throughout the growth period (data not shown).

### Effects of substrates on the morphology of free-living CzR2

**Fig. 5:**
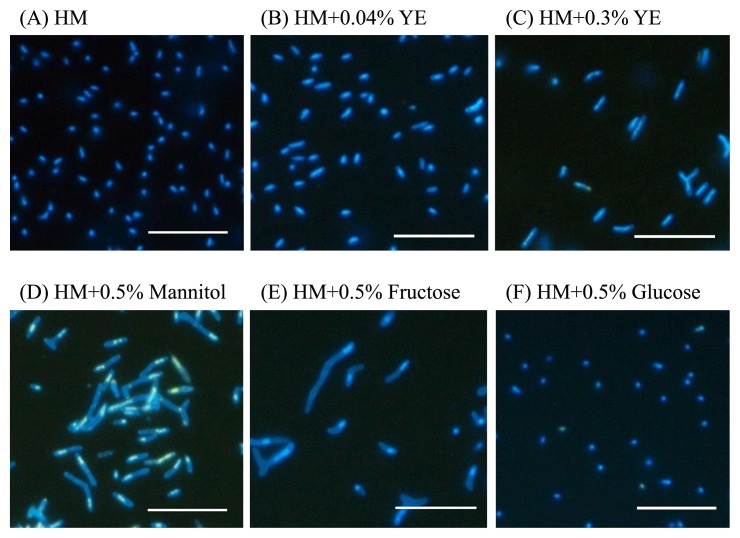
Morphology of strain CzR2 grown with different substrates. CzR2 was cultivated on plates of HM (HEPES-MES) basal medium (A), HM+0.04% yeast extract (YE) (B), HM+0.3% YE (C), HM+0.5% mannitol (D) HM+0.5% fructose (E), and HM+0.5% glucose (F) for 14 d. Cells were stained with DAPI, bar=10 μm.

Strain CzR2 was uniformly rod-shaped and approximately 1–2 μm in length on the basal HM plate (Fig. 5A). In the presence of 0.04% yeast extract, the cells of CzR2 were slightly elongated (Fig. 5B). When grown on basal HM plates with 0.3% yeast extract, most CzR2 cells remained uniformly rod-shaped, whereas their length significantly increased to approximately 2–3 μm (Fig. 5C). In contrast, CzR2 grown on the HM plate with 0.5% of mannitol or fructose exhibited pleomorphism, with some cells becoming markedly elongated to 10 μm and some branching (Fig. 5D, 5E). The polar distribution of DNA was also observed in elongated and branched cells. However, the addition of 5% of glucose to the HM basal plate did not change the cell morphology of CzR2 (Fig. 5F). In contrast, the cells of USDA 110 grown with any of the tested substrates were uniformly rod-shaped (data not shown).

### Development, anatomy, and bacteroids of CzR2-infected nodules

**Fig. 6:**
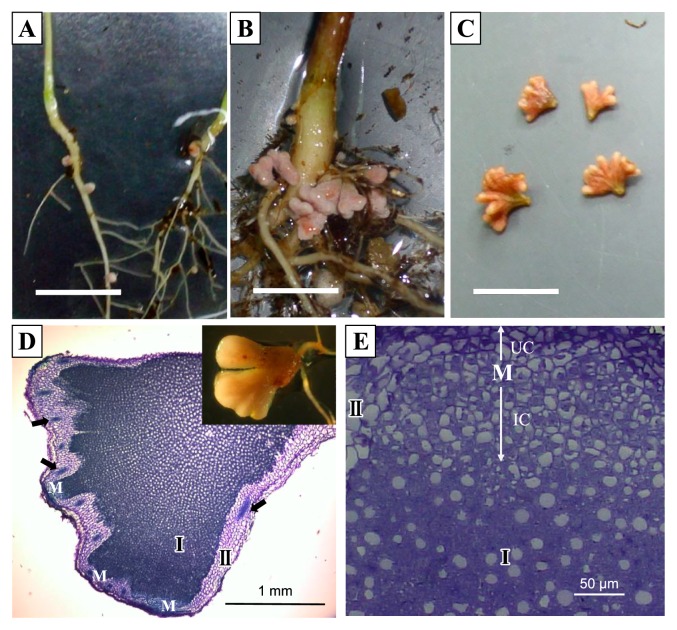
Nodulation characteristics of *C. zanzibarica* infected by strain CzR2. The nodules of *C. zanzibarica* inoculated by strain CzR2 were at 7 dpi (d post inoculation) (A), 35 dpi (B), and 56 dpi (C), bar=1 cm. Nodules were longitudinally sectioned and stained with toluidine blue O (D and E). The darkly-stained infection zone (I) was surrounded by a cortex (II) and vascular bundles (black arrow). The apical nodule meristem (M) showed two distinct cell groups; uninfected cells (UC) and infected cells (IC).

Strain CzR2 induced *C. zanzibarica* to form visible nodules (1 mm) 7 d post inoculation (dpi) (Fig. 6A). The nodules of seedlings at 35 dpi became branched (Fig. 6B), while those of plants at 56 dpi displayed a multi-lobed morphology (Fig. 6C). In contrast, no nodules were observed on the control plants without inoculation.

The anatomical features of the nodules were presented in Fig. 6D and 6E, and showed that all plant cells in the infection zone were uniformly infected and no infection thread was formed.

**Fig. 7:**
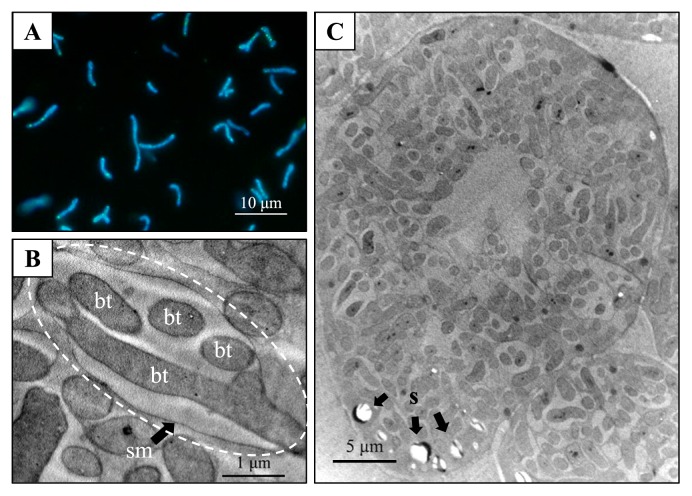
Cellular morphology of strain CzR2 symbiotic with *C. zanzibarica*. Bacteroids of strain CzR2 stained with DAPI displayed pleomorphism (A). TEM images of a root nodule of *C. zanzibarica* showed that a single symbiosome (dashed circle) was encompassed by the symbiosome membrane (sm) and harbored multiple bacteroids (bt) (B). Infected cells were filled with bacteroids, except for the central region and starch grains (s) scattered on the periphery (C).

Bacteroids extracted from the multi-lobed nodules of *C. zanzibarica* were highly pleomorphic and some were longer than 10 μm in length (Fig. 7A). TEM images of nodule sections also revealed that strain CzR2 displayed pleomorphism inside host cells. Additionally, symbiosomes commonly comprised multiple bacteroids (Fig. 7B, 7C).

### DNA content of free-living and symbiotic CzR2

**Fig. 8:**
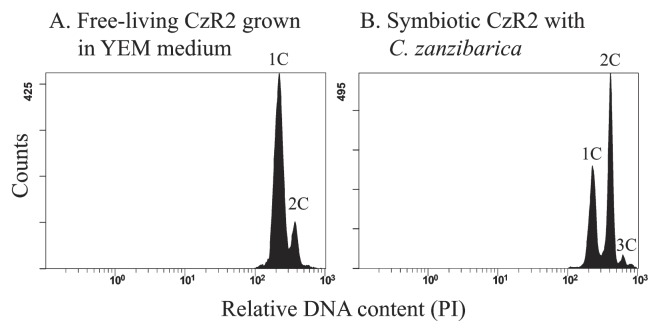
Flow cytometry analyses of DNA contents in free-living and symbiotic CzR2. Cells were stained with propidium iodide (PI). Free-living CzR2 (A) was grown in YEM broth and sampled from the late log phase, and bacteroids (B) were extracted from the nodules of 67-d-old *C. zanzibarica*. The x axis shows fluorescence levels, indicating DNA contents and the y axis shows cell counts. In each experiment, 20,000 cells were analyzed.

Histograms produced by a flow cytometric analysis were shown in Fig. 8. Two peaks (representing 1C and 2C) of DNA contents were detected in cells of free-living CzR2, while at least three peaks (representing 1C, 2C, and 3C) were found in symbiotic CzR2 isolated from the nodules of *C. zanzibarica*.

## Discussion

In the present study, we isolated the rhizobial strain, CzR2, from the roots of greenhouse-grown *C. zanzibarica* and found that this strain displayed a bacteroid-like morphology in its free-living state. To the best of our knowledge, this is the first study to show that free-living rhizobia exhibit pleomorphism in standard YEM medium. Based on the MLSA of six housekeeping genes, strain CzR2 was classified as *B. arachidis*. The housekeeping and symbiotic gene sequences of strain CzR2 and *B. arachidis* CCBAU 51107^T^ were nearly identical (>99% similarity). Wang *et al.* (36) previously reported that *B. arachidis* is a peanut symbiont in China, with rod-shaped cells that are between 1.30 and 1.97 μm in length and have a generation time of 8.8 h in YEM broth. Consistent with their findings, we showed that strain CzR2 had the ability to nodulate the peanut. However, the cell length (Fig. 3) and generation time (*ca.* 30.2 h) of CzR2 were significantly different from those of *B. arachidis* CCBAU 51107^T^ reported by Wang *et al.* (36). In order to identify the reason for this discrepancy, we purchased and cultured *B. arachidis* CCBAU 51107^T^ (LMG 26795^T^), and confirmed that this type strain also displayed pleomorphism in standard YEM medium (Fig. S1). Besides strain CzR2 and CCBAU51107, we found another *B. arachidis* strain, isolated from field-growing *C. zanzibarica*, which displayed pleomorphism on YEM medium (Fig. S1). These results further confirmed that the pleomorphic phenotype of CzR2 is a genuine feature rather than an artifact. The causes of the unusual phenomenon in CzR2 were then analyzed in the present study.

Bacteria typically maintain their morphological uniformity due to equal cell division (39). Strain CzR2, with no exception, was uniformly rod-shaped on the basal HM plate (Fig. 5A), but exhibited diverse cell shapes and sizes (pleomorphism) in YEM medium. A pure bacterial culture displaying pleomorphism has been regarded as the product of the old culture (composed of aging and dying cells) or contaminants (35). In order to clarify whether the pleomorphism of CzR2 on YEM medium was caused by the aforementioned reasons, we measured the population growth and size of cells during different phases. The results obtained revealed that strain CzR2 in YEM broth gradually increased its cell size at the early exponential phase (Fig. 4), namely, when cells were young and active. In addition, even when CzR2 was sub-cultured repeatedly or re-isolated from legume hosts, it consistently exhibited pleomorphism. In contrast, this unusual phenomenon was never observed in the reference strain USDA 110 under the same culture conditions. Accordingly, we concluded that the pleomorphism displayed by CzR2 on YEM medium was not caused by cell senescence or contamination. Elongated cells of free-living rhizobia have also been reported when cultured in medium with dicarboxylate or a high concentration of yeast extract (21, 26). In the present study, we found that yeast extract stimulated cell elongation and some degree of pleomorphism in CzR2 (Fig. 5C). However, fructose and mannitol induced strain CzR2 from uniformly rod-shaped cells to highly pleomorphic and elongated cells (Fig. 5), which has not been reported by previous studies. Thus, the pleomorphism displayed by CzR2 on YEM medium is induced by gradients of yeast extract and mannitol in the medium. This result also indicates that the cell morphology of CzR2 is affected by nutrient conditions. The transformation into elongated and branched cells with polarity and increments in the cell surface (Fig. S2) may enhance the absorption of nutrients by cells (28, 39).

When inoculated with CzR2, *C. zanzibarica* produced multi-lobe indeterminate nodules (Fig. 6C), which is consistent with the specific nodule type (crotalarioid) described by Corby (3). Furthermore, the infection zone of nodules only contained infected cells and an infection thread was never observed (Fig. 6D, 6E), suggesting that the nodulation process of *C. zanzibarica* did not involve a root-hair infection pathway. These results support the nodulation process of genistoid legumes (such as *Lupinus*, *Crotalaria*) commonly starting from crack or epidermal infections with a few infected cells dividing repeatedly to form a uniformly infected zone (27). However, we found that symbiosomes commonly contained multiple bacteroids with pleomorphism (Fig. 7) in *C. zanzibarica*-CzR2 symbiosis, which is distinct from model legumes. In the nodules of the pea, each symbiosome typically harbors a single, but pleomorphic (such as swollen, elongated, or branched) bacteroid. In contrast, in the nodules of the soybean, each symbiosome generally contains multiple bacteroids of a uniform rod shape (7, 14). Furthermore, the symbiosome features of *C. zanzibarica* differed from those of other genistoid legumes. For example, symbiosomes within the nodule cells of *Cyclopia*, *Cytisus*, *Genista*, and *Lupinus* generally contained a single bacteroid (4, 6, 8, 24).

In galegoid legumes, repeated DNA replication without cytokinesis results in polyploidy (genomic endo-reduplication) and the elongation of bacteria during bacteroid maturation (13). Although strain CzR2 formed filamentous cells in its free-living and symbiotic states, a flow cytometric analysis revealed that cells in the free-living state had 1 and 2 sets, while those in the symbiotic state had 1, 2, and 3 sets of DNA contents (Fig. 8). These results suggest that the elongated cells of strain CzR2 in its free-living state are due to a cell cycle delay, while those in its symbiotic state are from genomic endo-reduplication. Swollen and polyploid bacteroids have also been reported in some genistoid legumes (16). Furthermore, bacteroid differentiation in the legumes of the galegoid clade is controlled by the host plant (13, 34). One hypothesis regarding the benefit of this transformation for the host plant is that swollen bacteroids fix nitrogen more efficiently than non-swollen ones (15). Accordingly, the elongated bacteroids found in the nodules of *C. zanzibarica* may exhibit superior symbiotic performance, which contributes to the unusually high nitrogen content of the host.

## Conclusion

The rhizobium CzR2 isolated from the nodules of *C. zanzibarica* belongs to a *B. arachidis* strain. Pleomorphism in this strain in its free-living state may be induced by the addition of yeast extract, mannitol, or fructose, but not by glucose, to HM medium, thereby indicating that this phenomenon is substrate-dependent. CzR2 in its free-living state contained haploid and diploid cells, while that in symbiosis with *C. zanzibarica* was elongated with polyploidy, suggesting the occurrence of genomic endo-reduplication.

## Supplementary Information
