# Supplementary material for: Phylogenetic Identification, Phenotypic Variations, and Symbiotic Characteristics of the Peculiar Rhizobium, Strain CzR2, Isolated from Crotalaria zanzibarica in Taiwan
Source: Microbes Environ. 2016 Sep 29;31(4):410–7. doi: 10.1264/jsme2.ME16063 (PMC5158113; doi:10.1264/jsme2.ME16063)
Supplement: Supplementary file 1 [file 31_410_s1.pdf]

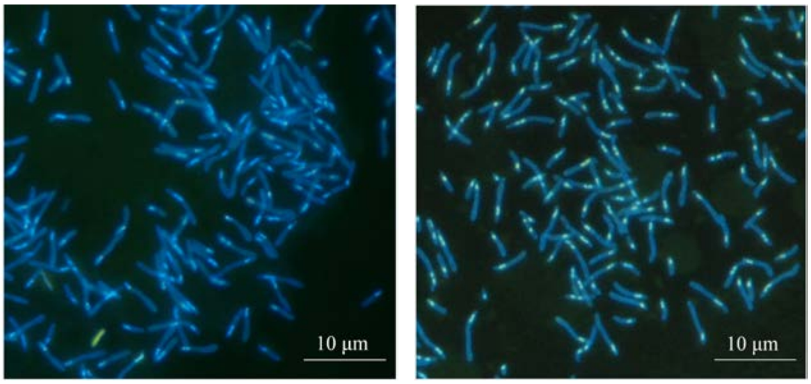

Fig. S1. Cellular morphology of *B. arachidis* CCBAU 51107^{T} (Left) purchased from BCCM (Belgian Coordinated Collections of Microorganisms) and strain CzHDF4 (Right) isolated from field-growing *C. zanzibarcia*. These strains were cultivated on YEM plate for 7 days at 30℃ and stained with DAPI.

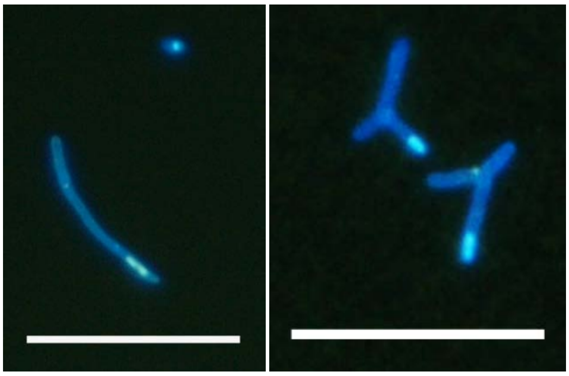

Fig. S2. Strain CzR2 displayed filamentous and branched cells. This cells were cultivated on YEM plate for 7 days at 30℃ and stained with DAPI. Bar = 10 μm.
